# Supplementary material for: Identification of photoperiod-induced specific miRNAs in the adrenal glands of Sunite sheep (Ovis aries)
Source: Front Vet Sci. 2022 Jul 22;9:888207. doi: 10.3389/fvets.2022.888207 (PMC9354845; doi:10.3389/fvets.2022.888207)
Supplement: Supplementary file 1 [file Data_Sheet_1.ZIP › Supplementary file/miRDeep2 file.zip!/miRDeep2 file/Know_Structure/SP42A3/oar-mir-432.pdf]

miRBase precursor	: oar-mir-432
Total read count	: 830
oar-miR-432 read count	: 829
remaining reads	: 1

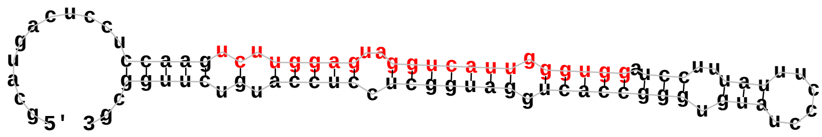

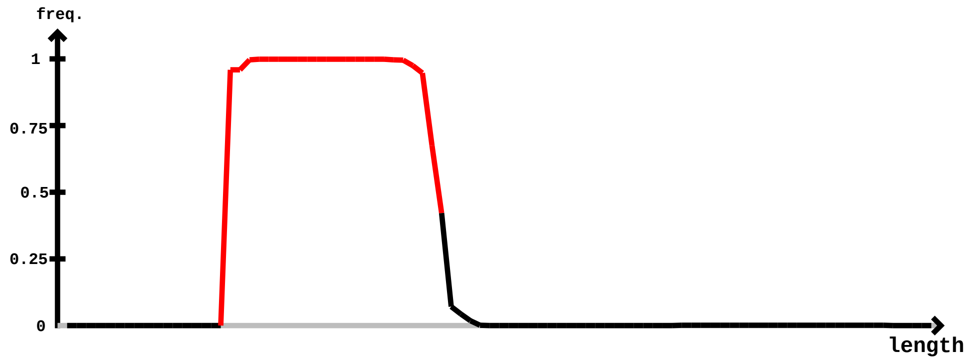

oar-miR-432

```
5'- gcaugacuccuccaagucuuggaguaggucauugggugggauccuuuauuucccuaugugggccacuggauggcuccuccaugucuuggcg   -3'    exp
    ...........(((((.(.(((((..(((((((.(((((.(((..(((......))).)))))))).))))))).))))).).)))))..    reads     mm        sample
    ................ucuuggaguaggucauu.........................................................    2         0         seq
    ................ucuuggaguaggucauug........................................................    1         0         seq
    ................ucuuggaguaggucauugg.......................................................    17        0         seq
    ................ucuuggaguaggucauuggg......................................................    23        0         seq
    ................ucuuggaguaggucauugggu.....................................................    226       0         seq
    ................ucuuggaguaggucauugggug....................................................    209       0         seq
    ................ucuuggaguaggucauuggguggg...................................................    290       0         seq
    ................ucuuggaguaggucauuggguggga..................................................    22        0         seq
    ................ucuuggaguaggucauuggguggau.................................................    5         0         seq
    ................ucuuggaguaggucauuggguggauc................................................    1         0         seq
    ..................uuggaguaggucauugggug....................................................    1         0         seq
    ..................uuggaguaggucauuggguggg...................................................    1         0         seq
    ..................uuggaguaggucauuggguggga..................................................    1         0         seq
    ..................uuggaguaggucauuggguggau.................................................    15        0         seq
    ..................uuggaguaggucauuggguggauc................................................    12        0         seq
    ..................uuggaguaggucauuggguggaucc...............................................    1         0         seq
    ...................uggaguaggucauuggguggau.................................................    1         0         seq
    ...................uggaguaggucauuggguggauc................................................    1         0         seq
    ...............................................................acuggauggcuccuccaugucu.....    1         0         seq
```
